# Supplementary material for: Can transcranial direct current stimulation (tDCS) over the motor cortex increase endurance running performance? a randomized crossover-controlled trial
Source: PLoS One. 2024 Dec 5;19(12):e0312084. doi: 10.1371/journal.pone.0312084 (PMC11620604; doi:10.1371/journal.pone.0312084)
Supplement: S2 Table — Adverse effects following active and sham transcranial direct current stimulation (tDCS). All adverse effects were considered as mild (i.e., did not require medical action). (PDF) [file pone.0312084.s002.pdf]

Adverse effects

Table S4: Adverse effects following active and sham transcranial direct current stimulation (tDCS).

Adverse effect	Active tDCS (n=45)	%	Sham tDCS (n=45)	%
Headache	2	4,4	4	8,9
Neck pain	0	0,0	2	4,4
Scalp pain	1	2,2	2	4,4
Tingling	25	55,6	24	53,3
Itching	7	15,6	4	8,9
Sleepiness	2	4,4	3	6,7
Trouble concentrating	5	11,1	6	13,3
Acute mood change	2	4,4	2	4,4
Other [a]	1	2,2	0	0,0

All adverse effects were considered as mild (i.e., did not require medical action).

[a] Sensation of heavy legs.
