# Supplementary material for: Can transcranial direct current stimulation (tDCS) over the motor cortex increase endurance running performance? a randomized crossover-controlled trial
Source: PLoS One. 2024 Dec 5;19(12):e0312084. doi: 10.1371/journal.pone.0312084 (PMC11620604; doi:10.1371/journal.pone.0312084)
Supplement: S1 File — (PDF) [file pone.0312084.s004.pdf]

UNIVERSITE DE LIEGE
Faculté de Médecine
Département des Sciences de la Motricité
Service de Physiologie de l'Effort Physique

Professeur T. Bury

Ergogenic and physiologic effects of transcranial direct current stimulation (tDCS) on maximal aerobic performance in recreational and trained runners: Protocol description

I. BACKGROUND AND SIGNIFICANCE

Performance encompasses several definitions within different contexts but in the sports area, the common thread to evaluate performance is either by one individual's *capacity* (i.e., the length of time that a given power output can be maintained) or by his/her *power* (i.e., the amount of time required to complete a given amount of work) (Coyle 1999). These two determinants are the basis for athletes' evaluation in competition, for training or for research studies.

The proportional influence of the different variables affecting performance has been a matter of debate for the past decades. Integrative models such as the one proposed by Coyle include, inter alia, the maximal oxygen consumption (VO_2max), the lactate thresholds or muscle typology (capillarity density, fiber composition…) (Coyle 1999). Saunders et al propose an even wider approach by including environmental factors (altitude, heat), anthropometry (limb morphology, bodyweight and composition) and training to the conventional physiologic factors (Saunders et al. 2004). These early models do not include the brain's participation in performing a prolonged exercise however many recent works emphasize the importance of central mechanisms in limiting performance (Marcora et al. 2009; Noakes 2011b, 2011a). For instance, the individual's motivation, perception of effort or previous knowledge of the effort can determine endurance performance (Pageaux 2014). Beyond motivation and more pragmatically, the cortical drive on the descending motor pathways directly affects the muscles' contractile ability and therefore the physical performance. The motor cortex (M1) is the most directly involved in exercise performance given its important neural drive on the motor neurons (Gandevia 2001). It has already been shown that muscle fatigue (i.e., the reduction in force/power generated by the muscle and induced by exercise) originates from both peripherals changes at the neuromuscular junction and supraspinal fatigue that directly arises from the failure from the motor cortex (M1) to generate force output through the motoneurons (Gandevia 2001; Taylor et al. 2016)). This is reflected by changes in cortical excitability in the motor cortex as measured by TMS-EEG (Taylor et al. 1996). Modulating this motor cortical excitability to increase the descending drive

to the motoneuron pool and thereby the motor output might be possible with transcranial direct current stimulation (tDCS).

tDCS is a non-invasive neuromodulation method using weak electrical current targeting specific cortical areas using electrodes applied on the scalp. tDCS uses direct current to modulate the excitability of the stimulated area and therefore the polarity of the current will influence its effects. The anode will have an excitatory effect by facilitating action potential release and by modifying the excitability of NMDA receptors, while the opposite inhibitory effects apply for the cathode (Stagg and Nitsche 2011). The effects of a single tDCS session of 9-13 minutes are transient and last about 60 to 90 minutes after the end of the stimulation (Nitsche and Paulus 2001). tDCS has already been used for a while in many different conditions and has now strong indications for neurological conditions such as chronic pain (Fregni et al. 2007), depression (Meron et al. 2015) and Parkinson (Doruk et al. 2014) but also for specific skills in both stroke and healthy populations such as hand dexterity (Au-Yeung et al. 2014; Pavlova et al. 2014), working memory (Ohn et al. 2008; Jo et al. 2009) and strength (Hummel et al. 2006; Williams et al. 2013). Therefore, the applications of tDCS are moving beyond therapeutic approaches for pathological populations to enhance cognitive and motor abilities in healthy subjects. For instance, reducing the perceived exertion during exercise (Okano et al. 2015) or enhancing decision-making (Ouellet et al. 2015).

Obviously, the field of exercise performance enhancement by tDCS has not been left behind and the amount of studies evaluating its effects on exercise performance has been gradually increasing since 2007, when Cogiamanian et al. investigated M1 tDCS-related changes in endurance time for a submaximal isometric contraction of left elbow flexors (Cogiamanian et al. 2007). This team was the first one to show that the time to exhaustion (TTE) was significantly longer following anodal tDCS applied over the right motor cortex. These findings on increased TTE were replicated afterwards with the interesting contribution that, in many cases, not only TTE was increased following M1 tDCS but the rate of perceived exertion increased more slowly, in the absence of effect on corticospinal excitability (Okano et al. 2015; Angius et al. 2016, 2018). This suggests the increase in TTE following stimulation is also due to the decreased perceived rate of exertion, which is known to be one of the most important features of fatigue (Noakes 2012).

A mini-review performed recently by Angius et al. 2016 identified 12 studies using tDCS to improve performance in either single or whole body dynamic exercise and underlines the heterogeneity in exercise types and tDCS set ups leading to mixed results (Angius et al. 2017). More recently, a systematic review with meta-analysis on the effects of tDCS on exercise performance has been performed and identified 22 studies, among which 11 could be included in the meta-analysis (Machado et al. 2019). It showed a significant effect favoring M1 anodal tDCS over sham tDCS only on cycling time to exhaustion (mean difference of 93.41 seconds). However, this was strongly influenced by a single study contributing for 84% of the weight. Looking at the 22 studies retrieved by this systematic review, the most striking observation is that none of them assessed tDCS effects on running performance. All the studies involving whole body dynamics focused on cycling only. This is surprising given time to exhaustion can be easily measured for several exercise modalities, including running; as many studies already showed (Hayes and Caplan 2014; Siler and Martin 2016; Martens et al. 2018). Additionally,

these treadmill tests to exhaustion do not only accurately reflect outdoor running conditions (Jones et al. 2015), they also elicit higher levels of VO_2max and increased TTE as compared to cycling on an ergometer, probably because the energetic demand for running is higher (by involving more muscular mass) (Billat et al. 1996a; Redkva et al. 2018).

Another observation is that these studies did not focus on measuring VO_2max, while it is a key physiological marker of performance. Indeed, as it represents the body's maximum capacity to use oxygen by delivering blood (cardiac output – also measured by heart rate) and consuming the oxygen (muscle metabolism), it is often used as a surrogate for an individual's aerobic capacity and is used in scheduling distance training (Billat et al. 1996b; Noakes 2000). During incremental exercises to exhaustion, VO_2max typically increases linearly with the intensity and then plateaus close to exhaustion (Taylor et al. 1955). In the same time, lactate accumulates in the blood compartment as a result of the glycolytic metabolic processes necessary to sustain muscles work. Therefore, quantifying this increase in blood lactate levels also accurately depicts the intensity of the exercise as well as the athlete's metabolic profile (Billat 1996; Saunders et al. 2004). Angius et al. showed a significantly greater accumulation of lactate in the blood compartment with bilateral M1 anodal tDCS, as compared to cathodal tDCS and sham (Angius et al. 2018)

Additionally, a variable that affects both the tDCS response and the performance is the athlete's baseline fitness level. Indeed, a previous study suggested individuals with a lower level of endurance capacity might benefit more from the ergogenic effects of tDCS (Williams et al. 2013) but this hypothesis still needs to be confirmed (Machado et al. 2019).

To summarize, while many different factors affect running performance, tDCS is a widely used and available tool and could hypothetically facilitate the supraspinal drive and thereby act upstream of the motor cortex to prolong the muscles work in time to exhaustion trials. While it appears to decrease the rating of perceived exertion (RPE) during cycling, little is known about the effects of tDCS on physiological performance parameters such as maximal oxygen consumption (VO_2max), maximal aerobic speed (MAS) and lactate thresholds in runners.

II. SPECIFIC AIMS

The aim of this prospective randomized sham-controlled clinical trial is to investigate the physiological effects of tDCS applied over the motor cortex on perceived exertion and performance-related parameters measured by an incremental treadmill test to exhaustion.
This research thus aims at answering the following questions:

1. Is active tDCS applied bilaterally over the motor cortex significantly more efficient than sham tDCS to improve the TTE?
2. Does active tDCS decreases the RPE, as compared to sham?
3. Is there any significant difference between active and sham tDCS on the physiological parameters measured during an incremental test to exhaustion, namely: VO_2max, MAS, respiratory exchange ratio, blood lactate levels, maximal heart rate?

4. Does the baseline level of physical fitness influences response to tDCS?

We hypothesize that 1) performance as measured by time to exertion will be increased following active and not sham tDCS due to a decreased perceived rate of exertion without significantly altering the other physiological parameters; and 2) performance as measured by incremental treadmill test to exhaustion will be more improved in recreational as compared to trained runners.

III. SUBJECTS

We will select 30 healthy subjects in each group (1: Trained; 2: Recreational, total of 60 subjects) who can provide informed consent. Subjects will be screened through the Exercise Physiology Laboratory database and within the University of Liège students pool. All participants will need to meet all the following inclusion criteria and none of the following exclusion criteria. Participants will be divided in two groups during the screening procedure: a beginners group (recreational runners) and a trained group. Criteria are as follows:

Inclusion criteria:

- Adult (18 – 35 years old)
- Male
- Free of any musculo-skeletal injury or pain for the past 6 weeks
- VO_2max between 30 and 40 mL/min/kg (beginners group) or between 40 and 60 mL/min/kg (trained group)
- Amount of training hours between 2 and 4 hours/week (beginners) or above 4 hours/week (trained)

Exclusion criteria:

- Pacemaker
- Intracerebral metallic implant
- Smoking
- Using dietary supplementation or medication potentially affecting the CNS
- Musculoskeletal injury within the past 6 weeks
- Presence of more than one ‘relative risk’ and/or 1 or more ‘high risk’ on the TSST (Transcranial direct current stimulation Safety Screening Tool) – Annex III

IV. STUDY PROCEDURES

This study is a double-blind sham-controlled study, using a crossover design. Each participant will undergo three sessions, separated by approximately 5 days: a baseline assessment including a questionnaire and an incremental treadmill test to exhaustion, an active tDCS session followed by a time-to-exhaustion trial and a sham tDCS session followed by a time-to-exhaustion trial. Active and sham tDCS sessions will be performed in a randomized order. The active session of

tDCS will consist in applying two anodes over M1 bilaterally (C3 and C4 according to the 10-20 international EEG placement) and the cathodes over the occipital areas (O1 and O2), and to inject 2 mA for 20 minutes through sponge electrodes (35 cm^2) placed on the scalp using the Startsim 8 (Neuroelectrics, Barcelona). The sham tDCS will use the same montage and device but direct current will only be injected for 30 seconds, with a ramp-up and ramp-down 15 seconds period, to mimic the somatosensory effects of active tDCS. The device offers a build-in double blind mode with a display depicting the exact same screen for active and sham stimulation. The randomization and programming of the device will be performed by a third party.

Visit 1: screening and baseline evaluation

Subjects will be initially screened using the laboratory database and the ones for whom meeting inclusion criteria is assumed will be contacted over the phone or in person to determine eligibility. They will be asked to refrain from vigorous activities and the ingestion of beverages containing caffeine and alcohol or of using tobacco for 24 h prior to each visit. The screening interview will be semi-structured using a questionnaire (see CRF) investigating the following:

- Demographic and contact information
- Training habits (hours, kilometers and number of sessions performed on a weekly basis)
- Last known VO_2max
- Life hygiene (smoking habits, dietary restrictions or supplementations, alcohol and caffeine consumption)
- History of musculoskeletal injuries or refractory pain
- Previous exposure to tDCS

The TSST (Transcranial direct current stimulation Safety Screening Tool) will also be administered (Bornheim et al. 2018) – See Annex I. If the subject scores ≥1 “Oui” for the part “Risque élevé” and/or >1 for “Risque relatif”, he will be excluded from the study.
If the subject meets all the inclusion criteria and none of the exclusion criteria at this stage, the following measurements will be performed.
The subject will be weighted, measured, and the body fat will be estimated from skinfold thickness using Durnin & Womersley’s method (Durnin and Womersley 1974). Baseline levels of peripheral oxygen saturation (SpO_2) and heart rate (HR) will be measured, using a pulse oximeter and a Polar belt, respectively.

The incremental treadmill test to exhaustion will be then be performed and will be similar as the one described in (Martens et al. 2018); the subject will warm up on the treadmill with 1% slope for 5 minutes at 8 km/h, then the pace will be incremented by 2 km/h every 2 minutes up to 16 km/h and then by 1 km/h every 3 minutes until the subject’s exhaustion (either volitional interruption or inability to keep the pace). To ensure reaching exhaustion, strong verbal encouragement will be provided and the maximal nature of the effort will be determined using the following criteria:

- heart rate above 90% of the age-predicted maximum (i.e. 220 – age);
- respiratory exchange ratio ≥ 1.10;

- plateau in VO_2max;
- lactate blood level ≥ 9 mmol/L.

This test will be performed in a temperature-controlled room.
Oxygen consumption, respiratory exchange ratio and heart rate will be measured continuously using an Ergostick system (Geratherm) and a Polar belt, respectively. Blood levels of lactate will be measured at the end of each increment using capillary blood collected on the subject's index and measured with a YSI 1500 Sport L-Lactate Analyser calibrated at 5 mmol/L.
The ratings of perceived exertion (RPE) will be assessed at the end of each increment using Borg's 15-grade scale, see Annex II (Borg 1998).

If the subject meets the remaining VO_2max criterion, he will move on to the next phase of the study with visit 2 and 3.

Visit 2 and 3: tDCS and post-tDCS evaluation

Each participant will be randomly assigned to receive either active tDCS on Visit 2 and sham tDCS on Visit 3 or the other way around, using a computer-generated randomization sequence.
At the beginning of each visit, SpO_2 and resting heart rate will be measured. tDCS will then be applied as described above. SpO_2 and resting heart rate will be measured right after tDCS and the subject will undergo a time-to-exhaustion treadmill test afterwards. This test will be performed at 90% of the maximal aerobic speed (MAS) until the subject's voluntary exhaustion or inability to keep the pace. The same variables as for Visit 1 will be measured continuously.
The experimental procedure is summarized in Figure 1.
At the end of each visit, two questionnaires will be administrated: the first one to assess if the subject thinks he received the active or sham tDCS, the second one to assess the presence of any adverse event, see Annex III (Brunoni et al. 2011).

Outcomes

Our primary outcome measure is the time to exertion (active vs. sham).
Our secondary outcome measures will be the changes pre-post tDCS in PRE, VO_2max, MAS, lactate thresholds, maximal heart rate, the 'pre' condition referring to the baseline assessment with the incremental test to exhaustion at Visit 1. Immediate physiological effects of tDCS will be evaluated using SpO_2 and HR measurements performed before and after each session.
These outcomes will be calculated at the group level and in the 2 groups separated based on baseline fitness level (beginners vs. trained).

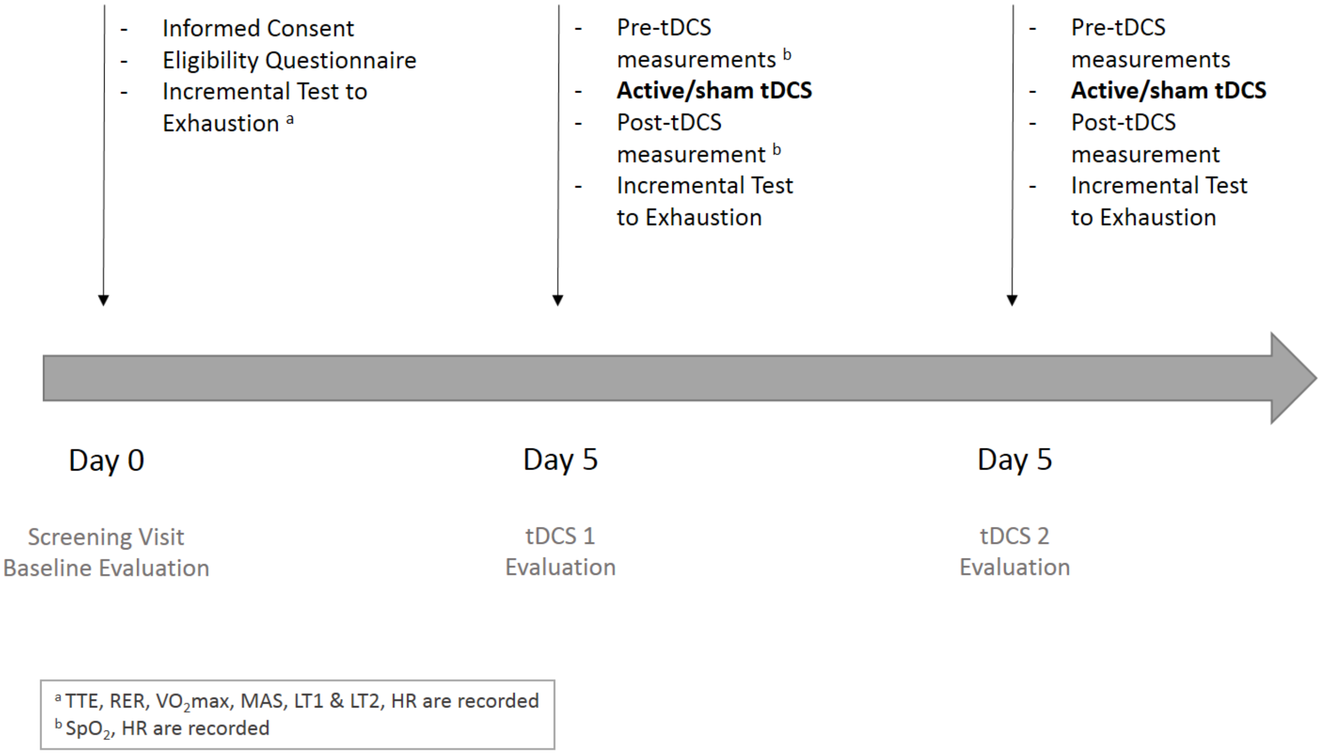

Figure 1: Experimental protocol

V. RISKS AND DISCOMFORTS

Transcranial Direct Current Stimulation (tDCS): This is a technique that poses a non-significant risk to subjects. The safety of this technique has been addressed and tested by multiple researchers (Nitsche et al. 2003a, 2003b, 2004; Iyer et al. 2005; Fregni et al. 2006) who have concluded that tDCS, applied in a manner similar to our proposed protocol, induces only temporary mood, cognitive/motor effects, and no negative side effects. More than 30 research studies involving hundreds of subjects have been published using tDCS. No undesirable or long-lasting effects have been reported, nor have any subjects reportedly abandoned a study due to discomfort. The most common side effects according to a recent consensus are: headache, dizziness, nausea, itchy sensation as well as irritation under the area of the electrodes (Nitsche, Cohen et al., 2008). Researchers at the National Institute of Neurological Disorders and Stroke (NINDS) (Iyer et al. 2005) conducted a safety study on tDCS, investigating 20-minute sessions of 1 mA and 2 mA current stimulation with healthy controls (n=103). No negative effects were identified. Additionally, Nitsche, Niehaus et al. found no measurable structural changes in brain tissue due to tDCS (Nitsche et al. 2004).

VI. REFERENCES

Angius L, Hopker J, Mauger AR. The ergogenic effects of transcranial direct current stimulation on exercise performance. Front Physiol. 2017;8.

Angius L, Mauger AR, Hopker J, Pascual-Leone A, Santarnecchi E, Marcora SM. Bilateral extracephalic transcranial direct current stimulation improves endurance performance in healthy individuals. Brain Stimul. 2018;11:108–17.

Angius L, Pageaux B, Hopker J, Marcora SM, Mauger AR. Transcranial direct current stimulation improves isometric time to exhaustion of the knee extensors. Neuroscience. 2016;339:363–75.

Au-Yeung SSY, Wang J, Chen Y, Chua E. Transcranial Direct Current Stimulation to Primary Motor Area Improves Hand Dexterity and Selective Attention in Chronic Stroke. Am J Phys Med Rehabil. 2014;93:1057–64.

Billat LV. Use of Blood Lactate Measurements for Prediction of Exercise Performance and for Control of Training. Sport Med. 1996;22:157–75.

Billat V, Faina M, Sardella F, Marini C, Fanton F, Lupo S, et al. A comparison of time to exhaustion at VO2max in elite cyclists, kayak paddlers, swimmers and runners. Ergonomics. 1996a;39:267–77.

Billat VL, Hill DW, Pinoteau J, Petit B, Koralsztein J-P. Effect of Protocol on Determination of Velocity at V̇O2max and on its Time to Exhaustion. Arch Physiol Biochem. 1996b;104:313–21.

Borg G. Borg’s Perceived exertion and pain scales [Internet]. Human Kinetics; 1998.

Bornheim S, Croisier JL, Maquet P, Kaux JF. Proposal of a new tDCS safety screening tool. Am J Phys Med Rehabil. 2018;1.

Brunoni AR, Amadera J, Berbel B, Volz MS, Rizzerio BG, Fregni F. A systematic review on reporting and assessment of adverse effects associated with transcranial direct current stimulation. Int J Neuropsychopharmacol. 2011;14:1133–45.

Cogiamanian F, Marceglia S, Ardolino G, Barbieri S, Priori A. Improved isometric force endurance after transcranial direct current stimulation over the human motor cortical areas. Eur J Neurosci. 2007;26:242–9.

Coyle EF. Physiological determinants of endurance exercise performance. J Sci Med Sport. 1999;2:181–9.

Doruk D, Gray Z, Bravo GL, Pascual-Leone A, Fregni F. Effects of tDCS on executive function in Parkinson’s disease. Neurosci Lett. 2014;582:27–31.

Durnin JVGA, Womersley J. Body fat assessed from total body density and its estimation from skinfold thickness: measurements on 481 men and women aged from 16 to 72 Years. Br J Nutr. 1974;32:77–97.

Fregni F, Boggio PS, Nitsche MA, Rigonatti SP, Pascual-Leone A. Cognitive effects of repeated sessions of transcranial direct current stimulation in patients with depression. Depress Anxiety. 2006;23:482–4.

Fregni F, Freedman S, Pascual-Leone A. Recent advances in the treatment of chronic pain with non-invasive brain stimulation techniques. Lancet Neurol. 2007;6:188–91.

Gandevia SC. Spinal and Supraspinal Factors in Human Muscle Fatigue. Physiol Rev. 2001;81:1725–89.

Hayes PR, Caplan N. Leg stiffness decreases during a run to exhaustion at the speed at VO2max. Eur J Sport Sci. 2014;14:556–62.

Hummel FC, Voller B, Celnik P, Floel A, Giraux P, Gerloff C, et al. Effects of brain polarization on reaction times and pinch force in chronic stroke. BMC Neurosci. 2006;7:73.

Iyer MB, Mattu U, Grafman J, Lomarev M, Sato S, Wassermann EM. Safety and cognitive effect of frontal DC brain polarization in healthy individuals. Neurology. 2005;64:872–5.

Jo JM, Kim YH, Ko MH, Ohn SH, Joen B, Lee KH. Enhancing the working memory of stroke patients using tDCS. Am J Phys Med Rehabil. 2009;88:404–9.

Jones AM, Doust JH, Jones AM, Doust JH. A 1 % treadmill grade most accurately reflects the energetic cost of outdoor running A 1 % treadmill grade most accurately reflects the energetic cost of outdoor running. J Sports Sci. 2015;0414:321–7.

Machado DG d. S, Unal G, Andrade SM, Moreira A, Altimari LR, Brunoni AR, et al. Effect of transcranial direct current stimulation on exercise performance: A systematic review and meta-analysis. Brain Stimul. 2019;

Marcora SM, Staiano W, Manning V. Mental fatigue impairs physical performance in humans. J Appl Physiol. 2009;106:857–64.

Martens G, Deflandre D, Schwartz C, Dardenne N, Bury T. Reproducibility of the Evolution of Stride Biomechanics During Exhaustive Runs. J Hum Kinet. 2018;64:57–69.

Meron D, Hedger N, Garner M, Baldwin DS. Transcranial direct current stimulation (tDCS) in the treatment of depression: Systematic review and meta-analysis of efficacy and tolerability. Neurosci Biobehav Rev. 2015;57:46–62.

Nitsche MA, Fricke K, Henschke U, Schlitterlau A, Liebetanz D, Lang N, et al. Pharmacological modulation of cortical excitability shifts induced by transcranial direct current stimulation in humans. J Physiol. 2003a;553:293–301.

Nitsche MA, Liebetanz D, Lang N, Antal A, Tergau F, Paulus W. Safety criteria for transcranial direct current stimulation (tDCS) in humans. Clin Neurophysiol. 2003b;114:2220–3.

Nitsche MA, Niehaus L, Hoffmann KT, Hengst S, Liebetanz D, Paulus W, et al. MRI study of human brain exposed to weak direct current stimulation of the frontal cortex. Clin Neurophysiol. 2004;115:2419–23.

Nitsche MA, Paulus W. Sustained excitability elevations induced by transcranial DC motor cortex stimulation in humans. Neurology. 2001;57:1899–901.

Noakes TD. Physiological models to understand exercise fatigue and the adaptations that predict or enhance athletic performance. Scand J Med Sci Sport. 2000;10:123–45.

Noakes TD. Is it Time to Retire the A.V. Hill Model? Sport Med. 2011a;41:263–77.

Noakes TD. Time to move beyond a brainless exercise physiology: the evidence for complex regulation of human exercise performance. Appl Physiol Nutr Metab. 2011b;36:23–35.

Noakes TD. Fatigue is a Brain-Derived Emotion that Regulates the Exercise Behavior to Ensure the Protection of Whole Body Homeostasis. Front Physiol. 2012;3:82.

Ohn SH, Park CI, Yoo WK, Ko MH, Choi KP, Kim GM, et al. Time-dependent effect of transcranial direct current stimulation on the enhancement of working memory. Neuroreport. 2008;19:43–7.

Okano AH, Fontes EB, Montenegro RA, De Tarso Veras Farinatti P, Cyrino ES, Li LM, et al. Brain stimulation modulates the autonomic nervous system, rating of perceived exertion and performance during maximal exercise. Br J Sports Med. 2015;49:1213–8.

Ouellet J, McGirr A, Van den Eynde F, Jollant F, Lepage M, Berlim MT. Enhancing decision-making and cognitive impulse control with transcranial direct current stimulation (tDCS) applied over the orbitofrontal cortex (OFC): A randomized and sham-controlled exploratory study. J Psychiatr Res. 2015;69:27–34.

Pageaux B. The Psychobiological Model of Endurance Performance: An Effort-Based Decision-Making Theory to Explain Self-Paced Endurance Performance. Sport Med. 2014;44:1319–20.

Pavlova E, Kuo M-F, Nitsche MA, Borg J. Transcranial direct current stimulation of the premotor cortex: Effects on hand dexterity. Brain Res. 2014;1576:52–62.

Redkva PE, Miyagi WE, Milioni F, Zagatto AM. Anaerobic capacity estimated by the sum of both oxygen equivalents from the glycolytic and phosphagen pathways is dependent on exercise mode: Running versus cycling. Martinuzzi A, editor. PLoS One. 2018;13:e0203796.

Saunders PU, Pyne DB, Telford RD, Hawley JA. Factors Affecting Running Economy in Trained Distance Runners. Sport Med. 2004;34:465–85.

Siler WL, Martin PE. Changes in Running Pattern during a Treadmill Run to Volitional Exhaustion: Fast versus Slower Runners. Int J Sport Biomech. 2016;7:12–28.

Stagg CJ, Nitsche MA. Physiological basis of transcranial direct current stimulation. Neuroscientist. 2011;17:37–53.

Taylor HL, Buskirk E, Henschel A. Maximal oxygen intake as an objective measure of cardio-respiratory performance. J Appl Physiol. 1955;8:73–80.

Taylor JL, Amann M, Duchateau J, Meeusen R, Rice CL. Neural Contributions to Muscle Fatigue: From the Brain to the Muscle and Back Again. Med Sci Sports Exerc. 2016;48:2294–306.

Taylor JL, Butler JE, Allen GM, Gandevia SC. Changes in motor cortical excitability during human muscle fatigue. J Physiol. 1996;490:519–28.

Williams PS, Hoffman RL, Clark BC. Preliminary Evidence That Anodal Transcranial Direct Current Stimulation Enhances Time to Task Failure of a Sustained Submaximal Contraction. Hug F, editor. PLoS One. 2013;8:e81418.

VII. ANNEXES

Annex I: TSST

	Oui	Non
**Risque élevé**		
1. Le sujet a-t-il déjà présenté des effets secondaires indésirables à la suite d'une application de tDCS?		
2. Le sujet présente-t-il des lésions cutanées ou un cuir chevelu sensible ?		
3. Le sujet souffre-t-il d'une maladie neurologique (par exemple: AVC, Parkinson, épilepsie, apraxie, tumeur, métastases, troubles vestibulaires ou vertige paroxystique, déficits moteur ou sensoriels, diminution importante de la vigilance ou de l'attention) ?		
4. Le sujet présente-t-il des déficits langagiers ou cognitifs sévères impactant sa capacité à fournir un consentement éclairé ? (Score en-deca de 18 au Mini-Mental State Examination)		
5. Le sujet a-t-il/a-t-il eu des implants dans le corps susceptibles d'être activés ou chauffés par un courant électrique (par exemple : pacemaker, dérivation intracrânienne, implants cérébraux métalliques tels que cochlée artificielle, électrodes de stimulation cérébrale profonde) ?		
6. Le sujet a-t-il tout autre matériel métallique dans la tête (par exemple : clips chirurgicaux, agrafes ou shrapnel) ?		
7. Le sujet souffre-t-il de migraines fréquentes ou intenses?		
**Risque relatif**		
8. Le sujet a-t-il déjà été victime d'un traumatisme à la tête ou subi une intervention neurochirurgicale au niveau cérébral ?		
9. Le sujet a-t-il déjà eu des complications médicales sérieuses, telles qu'une dysfonction pulmonaire, cardiaque, hépatique ou rénale ?		
10. Le sujet souffre-t-il d'une dépression légère ou sévère ?		
Si oui, suit-il un traitement médicamenteux ?		
11. Le sujet prend-t-il des agents psychotropes (par exemple : antiépileptiques, neuroleptiques, benzodiazépines ou antidépresseurs) ou avec un effet sur la plasticité cérébrale (par exemple : dopamine, fluoxetine ou D-amphetamine, inhibiteurs de canaux sodiques, calciques, antagoniste des récepteurs NMDA) ?		
12. Le sujet a-t-il un historique d'addiction alcoolique ou à d'autres substances?		
**Garder à l'esprit**		
13. Le sujet a-t-il déjà eu une IRM? Un EEG ? Une TMS ?		
14. ~~Si le sujet est de sexe féminin, est-il possible qu'elle soit enceinte~~ ?		

Annex II: Borg RPE 15-grade scale

« À quel niveau de pénibilité avez-vous perçu ce travail ? »

6	
7	TRES TRES LEGER
8	
9	TRES LEGER
10	
11	LEGER
12	
13	NI LEGER, NI DUR
14	
15	DUR
16	
17	TRES DUR
18	
19	TRES TRES DUR
20	

Annex III: tDCS Adverse Effects Questionnaire

Après la tDCS, éprouviez-vous un des symptômes ou effets secondaires suivants ?	Indiquez une valeur (1-4) dans l'espace ci-dessous (1, absent; 2, léger; 3, modéré; 4, sévère)	Si présent, est-ce relatif à la tDCS ? (1, pas du tout; 2, vaguement; 3, possiblement; 4, probablement; 5, certainement)	Notes
Mal de tête			
Douleur au cou			
Douleur au cuir chevelu			
Picotements			
Démangeaisons			
Sensation de brûlure			
Rougeur de la peau			
Somnolence			
Difficultés de concentration			
Changement d'humeur important			
Autre (spécifiez)			
