# Supplementary material for: Can transcranial direct current stimulation (tDCS) over the motor cortex increase endurance running performance? a randomized crossover-controlled trial
Source: PLoS One. 2024 Dec 5;19(12):e0312084. doi: 10.1371/journal.pone.0312084 (PMC11620604; doi:10.1371/journal.pone.0312084)
Supplement: S2 File — Number of participants per group according to power and effect size. (PDF) [file pone.0312084.s005.pdf]

Sample size estimation

The study sample size was estimated a priori based using G*power 3.1.9.2. based on previous studies [1–3] with amateur participants (cyclists and runners), the weighted mean difference (± SD) between active and sham tDCS was 1.36 ± 1.15 minutes. Based on studies [4–6] with competitive participants (cylists), the weighted mean difference (± SD) between active and sham tDCS was 0.29 ± 1.10 minutes. Based on this data, an effect size of 0.95 was calculated. A bilateral test of comparison of two independent means (Student's t-test) was then performed considering an alpha level of 5%, a power of 80% or 90% and varying the effect size from 0.1 (small effect) to 1.5 (large effect). The results for the different hypotheses are presented in Table S2. We based our estimation on an effect size of 0.8 (large effect) and of power of 80%: this meant including 26 subjects in each group (amateur and competitive) for a total of 52 subjects.

Table S2
Number of participants per group according to power and effect size

	Power	
	80%	90%
Effect size	n	n
0.1	1571	2103
0.2 (small effect)	394	527
0.3	176	235
0.5 (moderate effect)	64	86
0.7	34	44
**0.8 (large effect)**	**26**	**34**
0.85	23	31
0.90	21	27
0.95	19	25
1	17	23
1.5	9	11

References

[1] Angius L, Hopker JG, Marcora SM, Mauger AR. The effect of transcranial direct current stimulation of the motor cortex on exercise-induced pain. Eur J Appl Physiol 2015;115:2311–9. https://doi.org/10.1007/s00421-015-3212-y.

[2] Angius L, Mauger AR, Hopker J, Pascual-Leone A, Santarnecchi E, Marcora SM. Bilateral extracephalic transcranial direct current stimulation improves endurance performance in healthy individuals. Brain Stimul 2018;11:108–17. https://doi.org/10.1016/j.brs.2017.09.017.

[3] Baldari C, Buzzachera CF, Vitor-Costa M, Gabardo JM, Bernardes AG, Altimari LR, et al. Effects of transcranial direct current stimulation on psychophysiological responses to maximal incremental exercise test in recreational endurance runners. Front Psychol 2018;9:1–10. https://doi.org/10.3389/fpsyg.2018.01867.

[4] Barwood MJ, Butterworth J, Goodall S, House JR, Laws R, Nowicky A, et al. The Effects of Direct Current Stimulation on Exercise Performance, Pacing and Perception in Temperate and Hot Environments. Brain Stimul 2016;9:842–9. https://doi.org/10.1016/j.brs.2016.07.006.

[5] Okano AH, Fontes EB, Montenegro RA, Farinatti P de TV, Cyrino ES, Li LM, et al. Brain stimulation modulates the autonomic nervous system, rating of perceived exertion and performance during maximal exercise. Br J Sports Med 2015;49:1213–8. https://doi.org/10.1136/bjsports-2012-091658.

[6] Vitor-Costa M, Okuno NM, Bortolotti H, Bertollo M, Boggio PS, Fregni F, et al. Improving Cycling Performance: Transcranial Direct Current Stimulation Increases Time to Exhaustion in Cycling. PLoS One 2015;10:e0144916. https://doi.org/10.1371/journal.pone.0144916.
