# Supplementary material for: Can transcranial direct current stimulation (tDCS) over the motor cortex increase endurance running performance? a randomized crossover-controlled trial
Source: PLoS One. 2024 Dec 5;19(12):e0312084. doi: 10.1371/journal.pone.0312084 (PMC11620604; doi:10.1371/journal.pone.0312084)
Supplement: Attachment — Submitted filename: Response to Reviewers.docx [file pone.0312084.s007.docx]

We are grateful for the constructive criticism provided by the reviewers and appreciate the time commitment. We thank the editors and reviewers for the feedback and specific comments provided on our manuscript. We have addressed those as outlined below.

We hope this improved version will meet the Journal standards and meet the reviewers’ expectations.

Respectfully,

Géraldine Martens, on behalf of all authors

**Journal Requirements:**

When submitting your revision, we need you to address these additional requirements.

1. Please ensure that your manuscript meets PLOS ONE's style requirements, including those for file naming. The PLOS ONE style templates can be found at

https://journals.plos.org/plosone/s/file?id=wjVg/PLOSOne_formatting_sample_main_body.pdf and

https://journals.plos.org/plosone/s/file?id=ba62/PLOSOne_formatting_sample_title_authors_affiliations.pdf

We made sure that our manuscript meets PLOS ONE’s style requirements and made the necessary amendments, including those related to file naming and authors’ affiliations.

2. In the online submission form, you indicated that [The dataset will be made available by the corresponding author (G. Martens, geraldine.martens@uliege.be) upon motivated request.].

All PLOS journals now require all data underlying the findings described in their manuscript to be freely available to other researchers, either 1. In a public repository, 2. Within the manuscript itself, or 3. Uploaded as supplementary information.

This policy applies to all data except where public deposition would breach compliance with the protocol approved by your research ethics board. If your data cannot be made publicly available for ethical or legal reasons (e.g., public availability would compromise patient privacy), please explain your reasons on resubmission and your exemption request will be escalated for approval.

We have uploaded the dataset (excel file) as supplementary information and amended our statement on data availability accordingly.

3. Please include captions for your Supporting Information files at the end of your manuscript, and update any in-text citations to match accordingly. Please see our Supporting Information guidelines for more information: http://journals.plos.org/plosone/s/supporting-information.

We included captions for our Supporting Information files at the end of our manuscript and updated the in-text citations accordingly following the Journal guidelines.

4. We note that the original protocol that you have uploaded as a Supporting Information file contains an institutional logo. As this logo is likely copyrighted, we ask that you please remove it from this file and upload an updated version upon resubmission.

We removed the logo from the file and uploaded an updated version.

5. Please review your reference list to ensure that it is complete and correct. If you have cited papers that have been retracted, please include the rationale for doing so in the manuscript text, or remove these references and replace them with relevant current references. Any changes to the reference list should be mentioned in the rebuttal letter that accompanies your revised manuscript. If you need to cite a retracted article, indicate the article’s retracted status in the References list and also include a citation and full reference for the retraction notice.

We reviewed our reference list to ensure it is complete and correct and have not made any changes.

**Reviewers' comments:**

5. Review Comments to the Author

Reviewer #1: A randomized sham-controlled crossover trial was conducted which aimed to assess transcranial direct current stimulation (tDCS) efficacy on submaximal treadmill running time to exhaustion (TTE). TTE was not statistically different in the two groups.

Minor revisions:

1- Page 10, Section 2.3: State the statistical testing method which achieves 80% power.

We specified in the manuscript the statistical testing method. It now reads:

“The study sample size was estimated a priori using Student’s t-tests (see Supplementary Material) based on previous studies with amateur and competitive participants (cyclists and runners) [22,26,29–32].”

2- Section 2.7: The term "t-tests" is missing in the following sentence. “Comparisons between active and sham tDCS sessions were performed using paired Student’s [t-tests] and Wilcoxon tests.” Also specify the Wilcoxon tests: rank sum or signed rank.

Thank you. We corrected the sentence by adding the missing “t-tests”. We also specified the Wilcoxon tests used. This excerpt now reads:

“Baseline comparisons (age, weight, height, body fat, years of training, weekly training, VO2max, MAS, TTE on VO2max test, maximal heart rate, maximal respiratory exchange ratio) between groups (active/sham and sham/active) were performed using independent Student’s t-tests (normal distribution) and Wilcoxon rank-sum tests (non-normal distribution). Comparisons between active and sham tDCS sessions were then performed using paired Student’s t-tests and Wilcoxon signed-rank tests.”

3- To assist in the review process, add line numbering to the document.

We added line numbering to assist in the review process.

Reviewer #2: As you mentioned the most problem in your study, is single session intervention so it seems that one session in not enough to have impact in physical performance, so it is better to emphasize in the conclusion to consider this issue in further study and try to have more sessions in their studies.

We thank the reviewer for the feedback and fully agree with issue raised about the single session aspect. To increase transparency about it in our manuscript we added the following in the limitations section;

“Our main limitations pertain to the tDCS protocol. We used a single session delivered before the TTE trial for feasibility constraints, while the concurrent application of tDCS with the targeted activity may have been more efficient [18,56]. This method also prevents the investigation of the cumulative effects of tDCS using several consecutive sessions.”

As well as the conclusion:

“To conclude, no beneficial effect of M1-tDCS on running performance has been identified. The potential effect of multiple sessions remains unknown and warrants further research.”
